# Modelling Filler Dispersion in Elastomers: Relating Filler Morphology to Interface Free Energies via SAXS and TEM Simulation Studies

**DOI:** 10.3390/polym10040446

**Published:** 2018-04-16

**Authors:** Norman Gundlach, Reinhard Hentschke

**Affiliations:** School of Mathematics and Natural Sciences, Bergische Universität, D-42097 Wuppertal, Germany; n.gundlach@uni-wuppertal.de

**Keywords:** elastomers, lattice model, Monte Carlo simulation, surface tensions, small angle scattering, transmission electron microscopy

## Abstract

The properties of rubber are strongly influenced by the distribution of filler within the polymer matrix. Here, we introduce a Monte Carlo-based morphology generator. The basic elements of our model are cubic cells, which, in the current version, can be either silica filler particles or rubber volume elements in adjustable proportion. The model allows the assignment of surface free energies to the particles according to whether a surface represents, for instance, ‘naked’ silica or silanised silica. The amount of silanisation is variable. We use a nearest-neighbour site-exchange Monte Carlo algorithm to generate filler morphologies, mimicking flocculation. Transmission electron micrographs (TEM) as well as small angle scattering (SAS) intensities can be calculated along the Monte Carlo trajectory. In this work, we demonstrate the application of our morphology generator in terms of selected examples. We illustrate its potential as a tool for screening studies, relating interface tensions between the components to filler network structure as characterised by TEM and SAS.

## 1. Introduction

Polymer nanocomposites, i.e., polymer matrices containing nanoparticles of variable amounts and types, possess a broad range of applications [1]. In particular, nanofillers are standard ingredients of rubber compounds, most often added to improve mechanical toughness [2]. Because their relative amounts are rather high, the addition of filler does generally influence all properties—mechanical and others—to a significant extent. This also means that the addition of filler, its chemistry and processing alike can be used to adjust the material properties. Our focus is on rubber in tyre applications, where filler is added primarily as an reinforcing agent (e.g., [3]). It nevertheless affects other properties like rolling resistance, grip or wear (e.g., [4]). One key parameter in this context is dispersion [5].

Dispersion of filler involves the application of shear-forces to distribute filler uniformly in a polymer matrix. There are different levels of dispersion distinguished as visual, macro- and micro-dispersion. We concentrate on the latter—specifically on the dispersion ranging from primary particles over aggregates to the filler network on a scale of up to 1 μm. Even when the filler is uniformly dispersed in the elastomer matrix, the filler particles will tend to flocculate in the post-mixing stages like storage, extrusion or vulcanization [6,7,8,9,10,11] (similar structural developments can also be observed in other contexts like drying of polymer nanocomposites [12]). Our modelling approach to this phenomenon, discussed in the following, is driven by local equilibrium thermodynamics in conjunction with the interface tensions between the various components.

Experimentally different methods are employed to assess the dispersion of filler in a rubber matrix depending on the type of dispersion (e.g., [4]). In the case of micro-dispersion transmission electron microscopy (TEM), atomic force microscopy and small angle X-ray (SAXS) or neutron (SANS) scattering techniques are used—either individually or in combination [13,14,15,16,17,18,19,20,21,22,23]. In the following, our focus will be on the combination of TEM with SAXS.

Nanofillers in polymer matrices have been studied extensively using molecular dynamics (MD), Monte Carlo (MC) and related computer simulation techniques. Quantities of interest encompass polymer density profiles, polymer and polymer segment mobility, the effect of particles on the glass transition temperature or the system’s viscosity. A comprehensive overview is given in a recent article by Hagita et al. [24]. In the same reference, the authors study a particularly large system of ≈ 2000 nanoparticles embedded in ≈ 40,000 chains consisting of ≈ 1000 beads each. They compare two fixed nanoparticle configurations (dispersed vs. aggregated) when the simulation box is stretched. These large simulations involve quite rough coarse grained interaction potentials and are limited to very short times. A different type of simulation approach to the structure formation in nano-composites is described by Martin [25]. The studies described in this reference, as well as in the references therein, focus on the effects of grafted chains on the effective force between model nanoparticles in effective solvents and polymer melts, and yet another concept is developed in Ref. [26]. Structural information obtained via transmission electron microscopy and scattering methods is used to construct filler network structures, whose elastic properties are then investigated. Nevertheless, it was noted recently by Legters et al. [27] that there still is a large gap in our understanding of the complex hierarchical structures in the actual multi-component nano-composites in industrial applications. The focus of this work therefore is the dependence of the filler structure or morphology on the actual interfacial tensions of the real components, which is outside the usual quantities of interest mentioned above. An exception is the recent work by Stöckelhuber et al. [28]. They study filler flocculation in polymers in a simplified model derived from game theory, where nevertheless the interactions are based on interface free energies derived from thermodynamics. We shall return to this work below.

At the usual filler concentrations (volume fraction 10 to 20%), the dispersed aggregates have some contact with each other. These contacts play in important role in the pronounced nonlinearity exhibited by the dynamic moduli of filled elastomers (Payne effect). In previous work, we have modelled the contribution of single inter particle contacts in filler networks to energy dissipation, rolling resistance in particular [29], as well as reinforcement based on the chemical composition of the system [30]. Application of this approach on the macroscopic scale, particularly to the relation between molecular composition and dynamic moduli, requires information regarding the number of filler contacts along a load bearing network path (cf. [31]) and, generally speaking, the morphology of the network as a whole.

In this work, we discuss a filler morphology generator based on a coarse-grained description of the ingredients in conjunction with measured interface or surface tensions. We employ a nearest-neighbour site-exchange MC algorithm, where the transition probabilities are based on experimental interface tensions between three components (polymer, silica and silane), to model filler dispersion on the micro-scale. The basic elements of our model are cubic cells, which can be either silica filler particles or rubber volume elements. The model allows the assignment of different surface free energies to the particles according to whether a surface represents, for instance, ‘naked’ silica or silanised silica. The amount of silanisation is variable. Aside from the aforementioned motivation, the proposed morphology generator is useful for screening purposes, relating surface energies to filler structure.

## 2. Materials and Methods

### 2.1. Monte Carlo Flocculation

**Figure 1:**
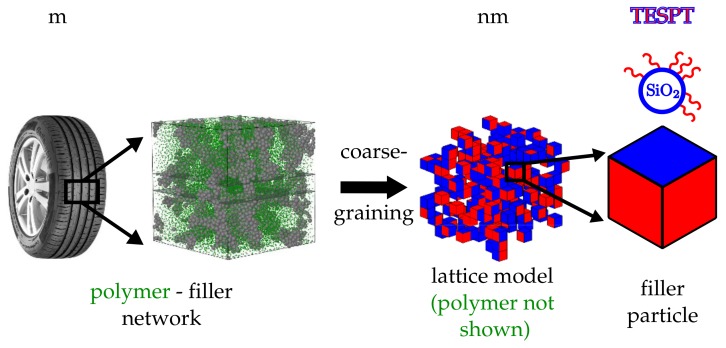
Hierarchy of scales. The polymer matrix within an elastomer composite, here exemplified by a tyre tread material, is reinforced by an embedded filler network. In the current model, the filler particles are approximated by cells on a cubic lattice (coarse graining). The different coloured faces are either representing bare particle surface areas (blue) or silanised areas (red). A specific example are silica particles silanised with TESPT.

Filler particles are modelled as cubic cells on an attendant lattice of size $L^{3}$. The property ‘filler’ initially is assigned to each cell on the lattice with probability ϕ. The remaining cells subsequently possess the property ‘rubber’. In principle, it is not difficult to introduce rubber blends. Here, however, we limit ourselves to just one type of polymer. Each of the six faces of a filler cell is silanised with probability θ. The remaining faces possess the property ‘bare silica’ or whatever the filler particles are made of. The third panel from the left in Figure 1 depicts a portion of such a system showing the filler particles only. Here, blue indicates a bare silica surface whereas red means that the surface is silanised.

**Figure 2:**
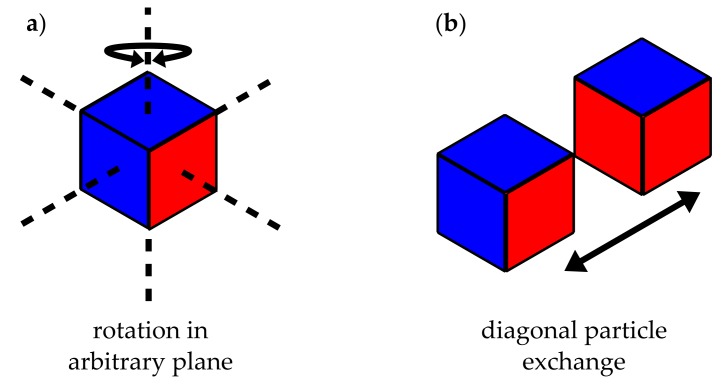
Illustration of MC moves. (**a**) particle (cube) rotation; (**b**) neighbouring particle exchange.

We model the flocculation process employing two local MC moves as depicted in Figure 2. The first move consists of the random selection of a lattice cell and its subsequent rotation by a random multiple of π/2 with respect to a likewise random axis of the lattice. Subsequently, a nearest-neighbour site exchange move interchanges two diagonal neighbour cells. Again, the pair to be exchanged is picked randomly. Note that these particular moves are chosen because they can be implemented quite efficiently. Each move separately is followed by a Metropolis criterion, i.e,

(1) min(1,exp[βΔW]≥ξ.

Here, $\beta^{-1}=k_{B}T$, where $k_{B}$ is Boltzmann’s constant and *T* is the temperature. In addition, ξ is a random number between zero and unity. If this inequality is satisfied, then the respective move will be accepted.

The quantity ΔW is obtained as follows. The equilibrium free enthalpy *G* of the system is given by

(2) $$\begin{array}{c} G=\frac{\sum_{i}G_{i}e^{-\beta G_{i}}}{\sum_{i}e^{-\beta G_{i}}}.\; \end{array}$$

The quantities $G_{i}$ denote the free enthalpies at fixed configurations *i*. Note that this simply follows from $\beta G=-\ln Q_{NPT}$ together with $Q_{NPT}=\sum_{i}Q_{i,NPT}=\sum_{i}e^{-\beta G_{i}}$ in conjunction G=N∂G/∂N and $G_{i}=N\partial G_{i}/\partial N$ (extensivity). In addition, at equilibrium

(3) $$dG{|}_{T,P,N_{k}}=\gamma_{j}dA_{j}.\;$$

Here, *P* is the pressure in the system, and $N_{k}$ is number of cells of type *k*. $\gamma_{j}$ denotes the interface tension of a face-to-face pairing of type *j* and $A_{j}=\;n_{j}\;a$ denotes the attendant total area of *j*-type interfaces in the system. Note that *a* is the effective contact area per face, which we assume to be the same for all *j*. Notice also that we use the summation convention. The proper ΔW, for a system developing towards equilibrium under NPT-conditions, and therefore is given by $\Delta W=-\gamma_{j}\Delta A_{j}$, i.e.,

(4) $$\exp\left[\beta \Delta W\right]=\exp\left[-\beta \;\gamma_{j}\;a\;\Delta n_{j}\right].\;$$

This generates system configurations satisfying Equation (2) on average. Away from equilibrium, the algorithm will drive the system towards the lowest possible free enthalpy *G* and the number of MC moves should be a rough measure of time. This may be justified by the local nature of the moves in conjunction with the assumption of a local equilibrium. The latter is commonly invoked during the derivation of transport equations in the framework of non-equilibrium thermodynamics [32].

### 2.2. Surface Tensions

Our thermodynamic modelling approach to flocculation is different from the game-theoretical algorithm proposed in Ref. [28]. However, as the authors of Ref. [28], we also model the particle-to-particle interaction in terms of interface tensions. The interface tensions $\gamma_{j}$ are expressed via the approximation

(5) $$\begin{array}{c} \gamma_{j}\equiv \gamma_{\alpha \beta }=\gamma_{\alpha }+\gamma_{\beta }-2\left(\sqrt{\gamma_{\alpha }^{d}\gamma_{\beta }^{d}}+\sqrt{\gamma_{\alpha }^{p}\gamma_{\beta }^{p}}\right). \end{array}$$

Note that $\gamma_{\alpha }=\gamma_{\alpha }^{d}+\gamma_{\alpha }^{p}$. The same applies to $\gamma_{\beta }$ of course. Here, the superscripts *d* and *p* indicate the dispersive and polar part of the surface tensions of α or β. Notice that a detailed discussion of this equation can be found in Ref. [33]. In addition, all numerical values for the surface tensions used in the following examples, unless stated otherwise, are taken from this work. It should be noted that the silane used throughout this paper is always precipitated silica, surface modified with TESPT (Coupsil 8113, powder form, $\gamma_{p}^{d}=22.2$ m$J/m^{2}$ and $\gamma_{p}^{p}=10.8$ m$J/m^{2}$) and that every fourth face is silanised, i.e., θ=0.25.

**Figure 3:**
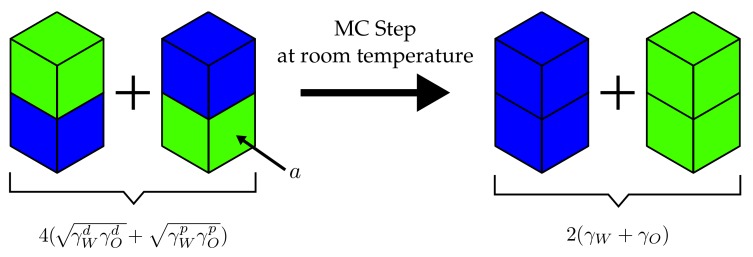
Example MC step in a water–oil mixture as explained in the text. The neighbouring particle exchange step is performed at room temperature, i.e., $k_{B}T=2.48$ kJ/mol. The interfacial area for each type of interface is *a*.

It is useful to consider the example depicted in Figure 3. The four cells, two corresponding to water and two corresponding to oil, initially possess two mixed interfaces for which j=wo. Subsequently, the cells are rearranged so that the water(w)–oil(o) interfaces are replaced by water–water (j = ww) and oil–oil (j = oo) interfaces. The attendant ΔW is given by

(6) $$\Delta W=-\gamma_{j}\;a\;\Delta n_{j}=-\gamma_{ww}a-\gamma_{oo}a+2\gamma_{wo}a.\;$$

Inserting Equation (5), we find

(7) $$\Delta W=2a\left(\gamma_{w}+\gamma_{o}-2\left(\sqrt{\gamma_{w}^{d}\gamma_{o}^{d}}+\sqrt{\gamma_{w}^{p}\gamma_{o}^{p}}\right)\right).\;$$

If we now use the values $\gamma_{w}^{d}=13.1$ kJ/(mol·nm${}^{2}$), $\gamma_{w}^{p}=30.7$ kJ/(mol·nm${}^{2}$) and $\gamma_{o}^{d}=18.9$ kJ/(mol·nm${}^{2}$), $\gamma_{o}^{p}=0.96$ kJ/(mol·nm${}^{2}$) (olive oil) [34], we find at room temperature, i.e., $k_{B}T=2.48$ kJ/mol

(8) $$\beta \Delta W\approx 17\;a\;\mathrm{nm}{}^{-2}.$$

This means that this particular MC step is accepted—regardless of what the concrete size of *a* is. In the following, we use experimental surface tensions obtained from literature sources.

### 2.3. Calculation of TEM Pictures and SAXS Intensities

**Figure 4:**
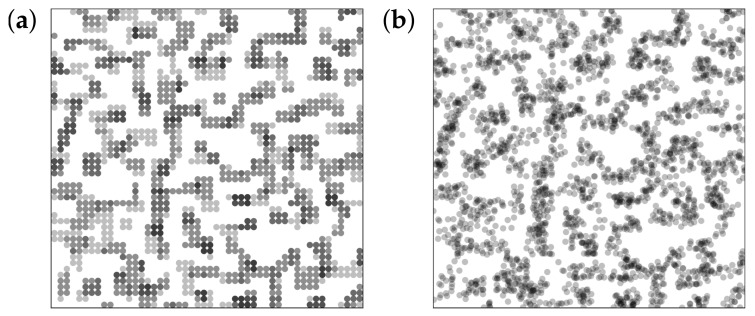
Mock TEM picture generation. (**a**) a slice, with a thickness of 5-particle diameters, is extracted from the simulation after a certain number of MC steps; (**b**) small random displacements, as described in the text, are applied to every particle in the slice. Darker spots are due to two or more particles superimposed along the line of sight. The polymer is polybutadiene rubber (BR, Lanxess Buna CB25, $\gamma_{p}^{d}=18.4$ m$J/m^{2}$ and $\gamma_{p}^{p}=3.7$ m$J/m^{2}$) and the filler precipitated silica (Ultrasil VN3, granulated form, $\gamma_{p}^{d}=18.7$ m$J/m^{2}$ and $\gamma_{p}^{p}=22.7$ m$J/m^{2}$). A filler volume content of ϕ=20% and a temperature of T=160 ${}^{\circ }$C were used.

Transmission electron micrograph images are generated from slices, five cells thick, extracted from the system after a certain number of MC steps. An example is shown in the left panel of Figure 4. Grey circles correspond to filler cells on the lattice. The shading becomes darker when filler cells are stacked along the line of sight. In order to increase the similarity to experimental TEM slices, it is useful to apply small random displacements to the circles. The maximum displacement in any direction is 0.6 times the lattice spacing (Note that the same procedure precedes the calculation of scattering intensities). Applying this to the aforementioned slice in Figure 4, we obtain the right panel.

**Figure 5:**
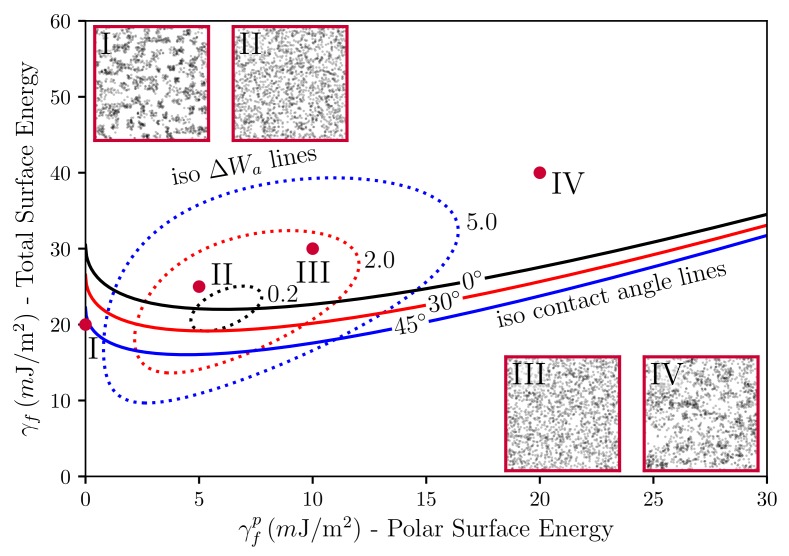
Plot of the polar part of the surface energy versus the total surface energy of the filler particles with constant wetting angles (solid lines), iso-$\Delta W_{a}$ lines (dotted loops), in units of m$J/m^{2}$, and TEM pictures. The dispersive part of the filler particles was kept fixed at $\gamma_{f}^{d}=20$ m$J/m^{2}$. Systems I to IV only differ in their $\gamma_{f}^{p}$-values. The polymer is natural rubber (NR, TMR—Standard Malaysian Rubber SMR 20, $\gamma_{p}^{d}=15.9$ m$J/m^{2}$ and $\gamma_{p}^{p}=6.1$ m$J/m^{2}$). Filler volume content is ϕ=15% and temperature is T=140${}^{\circ }$C.

Before we move on to the calculation of the SAXS intensities, we interject a brief discussion of our simulated TEM images in relation to the underlying surface tensions. Stöckelhuber et al. [35] have used a so-called wetting-envelope—work of adhesion plots to discuss polymer-filler compatibility and its dependence on the dispersive and polar parts of the surface tensions, respectively (see also Ref. [36]). Wetting-envelopes allow for representing regions of compatibility in the plane defined by the two surface tension components of one species keeping the surface tension components of the second species fixed. Figure 5 is an example of such a wetting-envelope—work of adhesion plot including simulated TEM pictures generated by the presented model. The solid lines are obtained via

(9) $$\begin{array}{c} \left(\gamma_{p}^{d}+\gamma_{p}^{p}\right)\frac{\cos\theta +1}{2}=\sqrt{\gamma_{p}^{d}\gamma_{f}^{d}}+\sqrt{\gamma_{p}^{p}\gamma_{f}^{p}}\;. \end{array}$$

Equation (9) is due to Owens and Wendt [37] (cf. Equation (7) in their paper), who developed it drawing on earlier work by Fowkes [38]. The subscripts *p* and *f* stand for polymer and filler, respectively. In their original work, Owens, Wendt and Fowkes focus on the wetting behavior of liquids on solid surfaces. Here, we equate the liquid with the polymer and the solid with the filler (note that in Ref. [36] the authors equate the liquid with the filler and the solid with the polymer. Notice also that Equation (9) is not symmetric with respect to this exchange.). The quantity θ is the contact or wetting angle of a liquid drop on a planar solid substrate in the well-known Young equation. The solid lines in Figure 5 are obtained by solving Equation (9) in the $\gamma_{f}^{p}$-$\gamma_{f}$-plane keeping the quantities $\gamma_{p}^{d}$, $\gamma_{p}^{p}$ and θ fixed. Increasing θ-values corresponds to decreasing wettability (or compatibility). In addition, the dashed closed loops in the figure are lines of constant work of adhesion between the polymer and the filler (for fixed values of $\gamma_{p}^{d}$ and $\gamma_{p}^{p}$). Note that work of adhesion, $W_{a}$, is defined as the free energy change, or reversible work, to separate a unit *p*-*f*-interface from contact to infinity (e.g., [39]), i.e., $W_{a}=\gamma_{p}+\gamma_{f}-\gamma_{pf}$. Here, we are interested in the change of the work of adhesion, $\Delta W_{a}$, which is given by $\Delta W_{a}=W_{a,pp}+W_{a,ff}-2W_{a,pf}$. This really is a difference of (reversible) works of adhesion, because the intermediate states, corresponding to the separated interfaces, cancel. $\Delta W_{a}$ is given by Equation (7) if we replace the subscripts w,o by f,p, respectively, and set *a* equal to the unit area. $\Delta W_{a}$ is the driving force for flocculation/reagglomeration according to Wang [40]. Moving away from the central loop means larger values of $\Delta W_{a}$ and a correspondingly stronger tendency for the filler to flocculate.

The four red dots in the $\gamma_{f}^{p}$-$\gamma_{f}$-plane indicate the surface tensions for which the TEM insets in Figure 5 were generated (each inset was obtained after 1000 MC steps per lattice cell (on average) in systems of size $128^{3}$). Notice that the insets II and III exhibit good dispersion in accordance with their position above the θ=0-line and their close proximity to the inner loop. Inset I, on the other hand, is far below the θ=0-line and also far from the inner loop. This is consistent with the apparent lumpiness of filler in this image. Inset IV is somewhat special. It is located above the θ=0-line but far from the inner loop. Again, we observe a lumpy filler distribution. However, the intra-filler interfacial structure differs very much from case I. Closer inspection reveals that intra-filler contacts in I are mostly between ‘naked’ filler faces, whereas, in IV, these intra-filler contacts are virtually absent. Instead, in IV, we observe largely intra-filler contacts between silanised faces of the cubic filler particles. The reader is reminded that the simulated systems discussed here contain filler particles represented by cubic cells where on average every fourth face is silanised. The above $\gamma_{f}$ refers to the naked filler surfaces. Silanisation in our model is an adjustable parameter. Thus, we can choose homogeneous silanisation, i.e., every face is silanised. However, a heterogeneous distribution of silane on the filler surfaces appears more realistic. Therefore, we have used the current example to highlight the possible importance of this point for the interpretation of wetting-envelope—work of adhesion plots.

In addition to the TEM images based on slices, we can compute the SAXS intensity based on the entire simulation box (see also [13]). The total intensity is the product of two factors, i.e.,

(10) $$I\left(q\right)=S_{a/n}\left(q\right)F_{P}\left(q\right).\;$$

The quantity *q* is the momentum transfer, i.e., the magnitude of the scattering vector. The form factor $F_{P}\left(q\right)$ is contributed by the filler (primary) particles. We do not model them explicitly. Instead, the particles are assumed to possess radial symmetry and a well defined surface. This means that, in the respective limits of small and large *q*, we have

(11) $$F_{P}\left(q\right)\propto \left\{\begin{array}{cc} S\;q^{-4}, & q\to \infty ,\\ V^{2}, & q\to 0. \end{array}\right.\;$$

Here, $S=4\pi R^{2}$ and $V=4\pi R^{3}/3$ are the surface and the volume of the particles, respectively. The $q^{-4}$-behaviour is known as Porod’s law [41]. For particles possessing a fractal surface structure, characterised by a surface fractal exponent $d_{s}$, one can show [42] that Porod’s law is replaced by $q^{-6+d_{s}}$. The cross-over from one limit to another occurs in a narrow regime around qR=π. This means that scattering intensity above the particular value of *q* is essentially constant and does not affect the *q*-dependence of the total intensity in this range. The latter is governed by $S_{a/n}\left(q\right)$, which is due to aggregated particles and the filler network in general. We express $F_{P}\left(q\right)$ in terms of an approximation due to Beaucage [43], combining the laws of Guinier and Porod, which is approximately valid over the entire *q*-range, i.e.,

(12) $$F_{P}\left(q\right)=\Delta \rho^{2}\left(V^{2}\exp\left[-q^{2}R^{2}/5\right]+2\pi Sq*{}^{-4}\right).\;$$

Note that $q*=q/{\left(erf\left(qR/\sqrt{10}\right)\right)}^{3}$ and Δρ is the contrast difference between filler and polymer. Realistic filler particles are polydisperse. Therefore, *R* is the mean particle size of the attendant distribution and $F_{P}\left(q\right)$ is the corresponding average intensity.

The structure factor $S_{a/n}\left(q\right)$, on the other hand, is given by

(13) $$S_{a/n}\left(q\right)=\phi \left(1+4\pi \rho \int_{0}^{\infty }drr^{2}\frac{\sin qr}{qr}\left(g_{2}\left(r\right)-1\right)\right).\;$$

**Figure 6:**
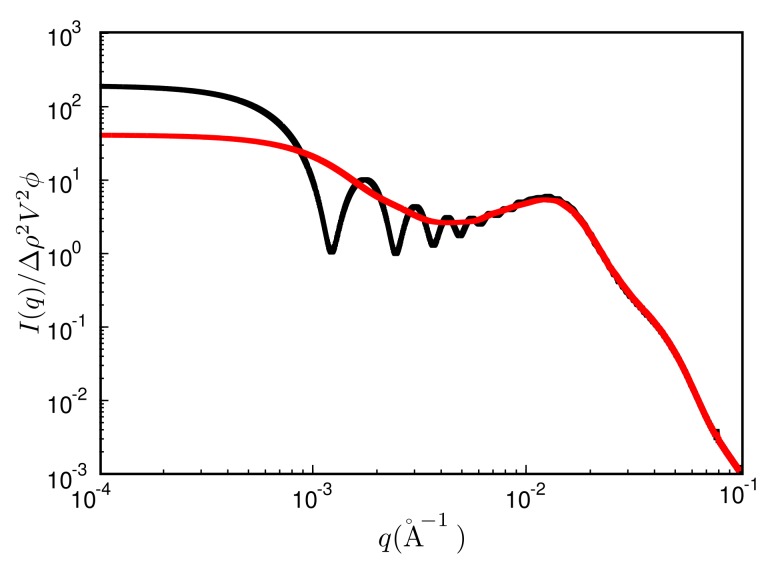
Reduced scattered intensity vs. magnitude of the scattering vector, *q*. Black: result obtained from a single MC configuration generated in a cubic box with periodic boundaries; red: result obtained after the averaging procedure explained in the text. System ingredients are the same as in Figure 4 but with a filler content of ϕ=15%. The temperature is T=160${}^{\circ }$C.

The quantity ρ is the filler particle number density and $g_{2}\left(r\right)$ is the radial filler particle pair-correlation function. Note that the upper bound of the integral is limited by the size of the simulation box. This leads to significant oscillations over a wide range of *q*-values (unless of course $g_{2}\left(r\right)=1$, which almost never is exactly true). Figure 6 shows an example, where the black curve is the reduced intensity obtained from a single finite box. The resulting oscillations may be reduced by averaging the intensities obtained for boxes of different size—akin to the primary particles themselves. This can be achieved for instance by repeatedly cutting smaller boxes from a single large simulation box at a given configuration. We use 30 to 50 such boxes varying in size between 100% to 18% in *L*, where $L^{3}$ is the volume of the original box. The result is the red curve in Figure 6. Note that the curve now is much more smooth, but the intensity is reduced in the small *q* limit. We return to this point below.

It is worth noting that the underlying length scales in Equations (12) and (13) are conceptually different. The length scale in Equation (12) is *R*, whereas in Equation (13), it is the lattice spacing, *d*. The simplest choice, which also yields the best results, amounts to setting R=d.

## 3. Results

**Figure 7:**
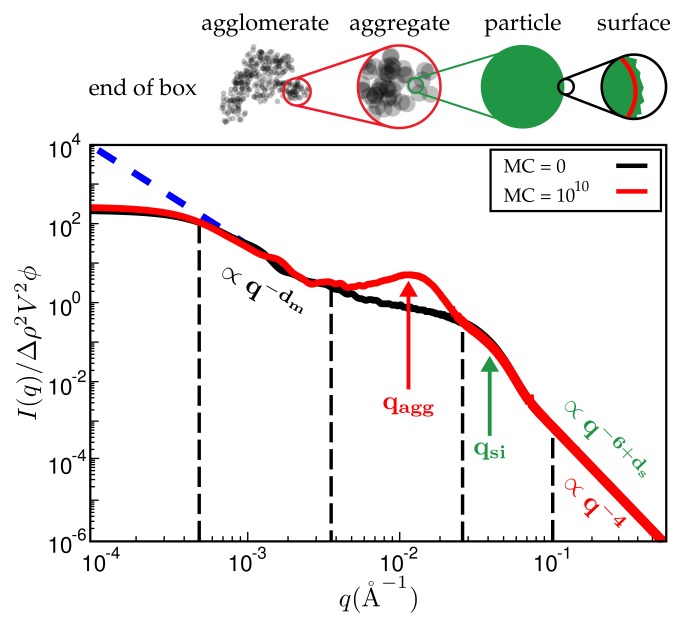
Reduced scattered intensity vs. magnitude of the scattering vector, *q*. The figure depicts the different *q*-regimes. The limit of large *q* is governed by Porod’s law or, in the case of fractal particle surfaces, by the attendant law exhibiting a fractal dimension. Subsequent *q*-regimes contain information on the size of the particles, their aggregates and the filler network itself. Due to the finite size of the simulation cell, there is a plateau terminating useful information at small *q*. The observed structure of course depends on the number of MC steps. System ingredients used here are the same as in Figure 4. The filler volume content is ϕ=20% and temperature is T=160${}^{\circ }$C.

Figure 7 is a typical plot of reduced intensity vs. *q* obtained at different stages of the MC. In the limit of small *q*, the intensity is governed entirely by $F_{P}\left(q\right)$ and thus is not affected by the MC at all. The average diameter of the primary filler particles, here $\approx 2\pi /q_{Si}$, is an input parameter and allows for expressing *q* in units of a specific inverse length. The strongest effect is due to formation of aggregates during the MC, leading to a peak that characterises the average aggregate diameter ($\approx 2\pi /q_{agg}$). The *q*-range labeled $\propto q^{-d_{m}}$ in Figure 7 reflects the super-structure beyond the aggregates. We expect this structure to be characterised by its mass fractal dimension $d_{m}$, i.e., the intensity in this regime should be $\propto q^{-d_{m}}$. The close to homogeneous initial filler distribution yields $d_{m}=3$. If the mixing produces a fractal network, we expect smaller values. The problem is that the attendant *q*-range should be at least an order of magnitude wide. This requires quite large system sizes of up to $10^{8}$ cells in our case. If *q* becomes very small, the box size eventually is exceeded and the scattering intensity levels off.

Even though Figure 7 is meant to illustrate the typical features of the simulated SAXS intensities, it is already in accordance with, for instance, SAXS intensities obtained by Schneider [13]. His Figure 7.15 shows the scattering intensity for natural rubber containing 20% (vol.) silanised silica (Ultrasil 7000/Si69 (bis(triethoxysilylpropyl)polysulfide)). The two humps, indicating the particle and the aggregate sizes, are clearly discernible, albeit less pronounced. The ratio $q_{si}/q_{agg}$ is about 3.5, which is comparable with the value in our Figure 7, i.e., 3.0. Of course, a more detailed comparison requires more careful attention to the surface tensions of the components and their relative abundances.

**Figure 8:**
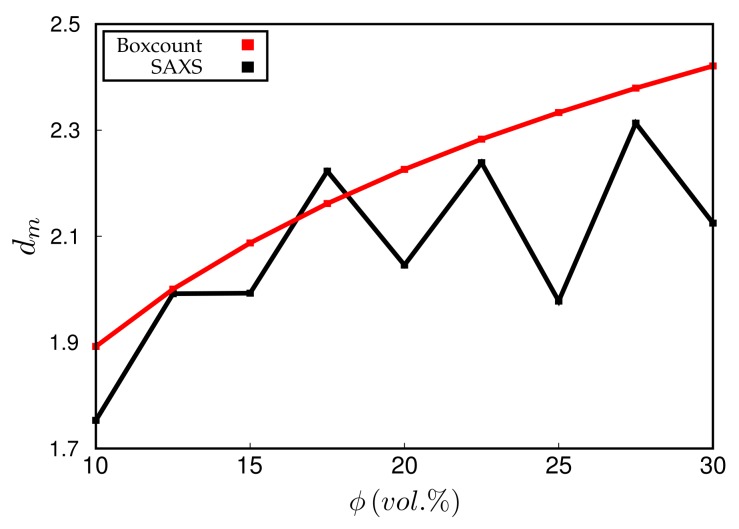
Mass fractal dimension, $d_{m}$, vs. filler volume fraction, ϕ. Black: $d_{m}$ calculated from fits to the scattering intensity in the range $5\times 10^{-4}<q<4\times 10^{-3}$; red: $d_{m}$ calculated via box-counting algorithm. Note that the system studied here for ϕ=20% is identical to the systems in Figure 4 and Figure 7.

Experimentally, $d_{m}$ is obtained via the slope of logI vs. logq at small *q*. Simulated scattering intensities can be analysed analogously. However, both the limited system size as well as the average over a distribution of boxes of variable size can and will affect the value of $d_{m}$. However, in the simulation, $d_{m}$ can be also measured directly using, for instance, a box-counting algorithm. This means that the system is partitioned into $n^{3}$ cells. Whenever a cell contains at least one particle, it is considered occupied. Plotting the logarithm of the number of occupied cells, $\ln A_{n}$, vs. lnn, should yield a slope equal to $d_{m}$ for sufficiently large *n*. Figure 8 compares the values of $d_{m}$ obtained by both methods for systems containing different amounts of filler. Notice that the system size is quite large in this case, i.e., the lattice dimension is 256×256×256. The numerical uncertainty of both methods is comparable, even though the box-counting algorithm appears to be smooth. The averaging over boxes of different size, as explained in the context of Figure 7, tends to reduce the slope of the scattering intensity in the *q*-regime where $d_{m}$ is determined. This is why the box-counting algorithm yields somewhat large values for the fractal dimension. In a recent work by Mihara et al. [20], the authors study flocculation in silica-filled rubber using small-angle X-ray measurements. Their fractal dimensions tend to be larger than the ones obtained here. For instance, using the conventional silica VN3, their $d_{m}$ increases from about 2.6 to 2.7 when the silica content increases from 60 to 80 phr. This corresponds to ϕ being roughly between 15% and 20% (vol.) in our case and thus the increase at least is comparable. Nevertheless, it is difficult to compare this conclusively because the general system compositions differ. Still, it is worth mentioning that Schneider [13] obtains mass fractal dimensions in the aforementioned system of U7000/Si69 (cf. the above discussion of Figure 6) in natural rubber between 2.5 and 2.6 when the filler content is about 20% (vol.). Mass fractal dimensions comparable to ours, i.e., around 2.3, are obtained by Koga et al. [16], albeit for (styrene-random-butadiene) copolymer (SBR) as well as polyisoprene (PI) loaded with 20% (vol.) carbon black.

**Figure 9:**
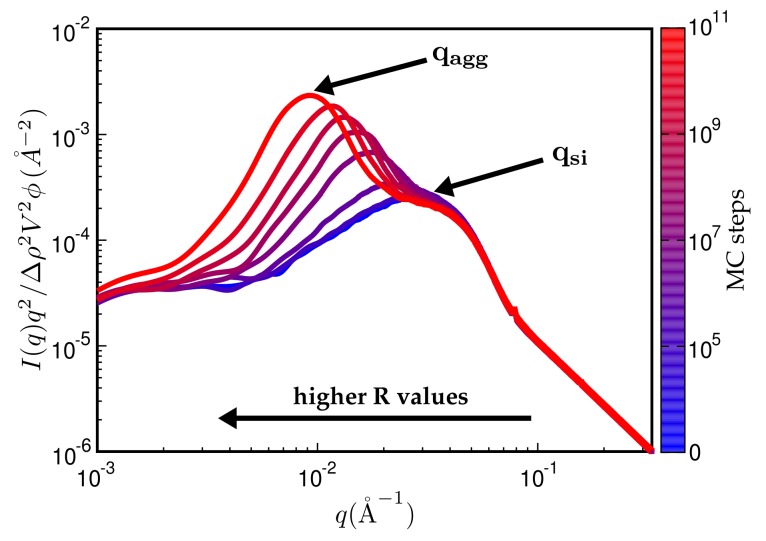
Kratky representation of the scattering intensity vs. *q* for different number of MC steps. An increasing number of MC steps shifts the aggregate peak at $q_{agg}$ to smaller *q* values, resulting in growing aggregates. The particle peak at $q_{si}$ remains at its position. The polymer is carboxylated acrylonitrile-butadiene rubber (XNBR, Lanxess Krynac X740, $\gamma_{p}^{d}=17.1$ m$J/m^{2}$ and $\gamma_{p}^{p}=33.3$ m$J/m^{2}$) and the filler fumed silica (Aerosil 200, Degussa, $\gamma_{p}^{d}=20.0$ m$J/m^{2}$ and $\gamma_{p}^{p}=17.3$ m$J/m^{2}$). Filler volume content is ϕ=25% and the temperature again T=160${}^{\circ }$C.

In the following, we discuss a number of examples illustrating the approach. In order to study aggregate formation, it is useful to multiply the scattering intensity by an extra factor $q^{2}$ (Kratky-plot). Figure 9 shows the reduced intensity using this Kratky-representation. Notice that the height of the aggregate peak increases and also shifts to smaller *q*-values with an increasing number of MC steps. At the beginning of the MC, only the particle-peak is present. Subsequently, the MC generates continuously growing aggregates for this particular system.

**Figure 10:**
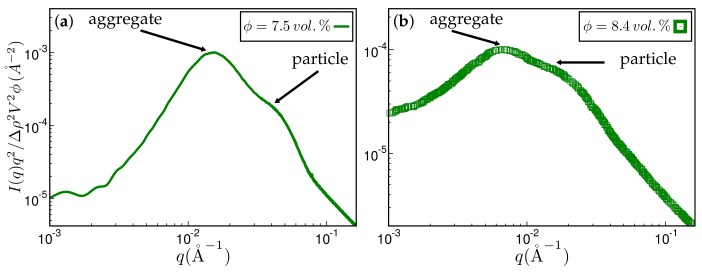
Approximate comparison between simulation and experiment. (**a**) Kratky representation of the scattered intensity obtained via simulation after $10^{10}$ MC steps on a 256 × 256 × 256 lattice. The system has the same ingredients as in Figure 4. The filler volume fraction is ϕ=7.5% and the temperature T=160 ${}^{\circ }$C. The mean particle size quoted in the literature is 〈R〉=80 Å. The aggregate size corresponding to the aggregate peak is about 202 Å; (**b**) experimental scattering curve taken from Ref. [19] for styrene-butadiene rubber (SBR) filled with Zeosil 1165 MP. The filler volume fraction is ϕ=8.4% and the temperature used to create this compound is T=160${}^{\circ }$C. The particle and aggregate sizes according to the attendant peaks are 139 Å and 402 Å, respectively.

The next figure, Figure 10, compares reduced scattering intensities obtained for related systems via simulation and experimental measurement. We note that the direct comparison between simulation and experiment thus far has been hampered by a lack of simultaneously available surface tension data, needed as input to the simulation, and attendant experimental SAXS intensities. Nevertheless, both simulation and experimental intensities are quite similar. A pronounced shoulder on the high *q* side indicates the primary particles. The shoulder is preceded by the aggregate peak, which, for the simulated system, is more pronounced. The ratio between aggregate and particle size is quite similar for both the experimental and the simulated system, i.e., $q_{si}/q_{agg}\approx$ 2.9 and 2.5, respectively.

**Figure 11:**
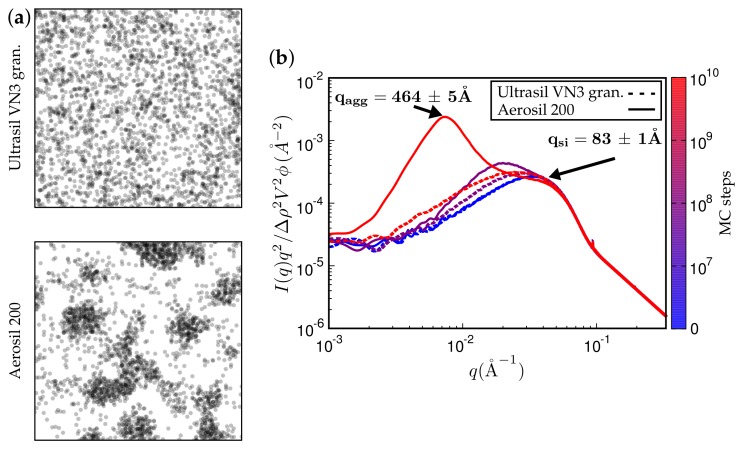
(**a**) simulated TEM images. (**b**) attendant simulated SAXS curves. The rubber is polychloroprene rubber (CR, Lanxess Baypren, $\gamma_{p}^{d}=19.3$ m$J/m^{2}$ and $\gamma_{p}^{p}=23.7$ m$J/m^{2}$) at 80% (by volume). Each system is either filled with 15% Ultrasil VN3 gran. at T=160${}^{\circ }$C (top TEM and dashed SAXS curves) or, alternatively, with Aerosil 200, thus leading to a silane content of 5% (by volume).

**Figure 12:**
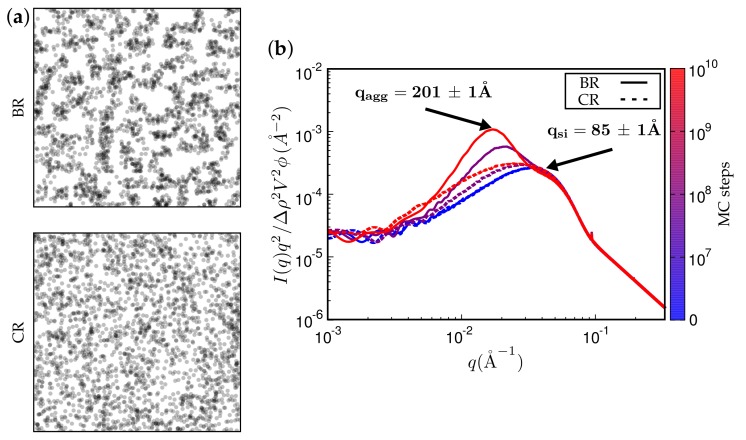
(**a**) simulated TEM images. (**b**) attendant simulated SAXS curves. Here, the filler is Ultrasil VN3 gran., as in one of the systems in the previous figure. The rubber is alternatively BR or CR. System parameters were not altered.

Figure 11 and Figure 12 present TEM and SAXS results in conjunction. Figure 11 compares two systems distinguished by different type of filler while keeping the remaining system parameters fixed. The TEM images correspond to the SAXS curves based on the maximum number of MC steps indicated. Notice that Ultrasil remains well dispersed, exhibiting little tendency for aggregation during the entire MC. Aerosil on the other hand, in this system, forms pronounced lumps of particles, which continue to increase during MC. Figure 12 shows what happens when the filler is Ultrasil in both cases, but the polymer is different—here polychloroprene rubber (CR, Lanxess Baypren) in comparison with BR. Very little aggregation is observed in CR, whereas, over the course of the indicated number of MC steps, small aggregates do form in BR. Their characteristic size is slightly larger than twice the size of the primary particles.

## 4. Discussion

We have developed a MC-based algorithm for the study of flocculation in filled rubbers in terms of TEM images and SAXS intensities in relation to the structural evolution of the filler distribution. The governing quantities are the interfacial free energies. Variable parameters include the amount of filler, the surface coverage with a compatibiliser, and the relevant interface tensions. TEM images and attendant SAXS curves are calculated along the trajectory, allowing the comparison to corresponding experimental systems. We also show in one example how wetting-envelope—work of adhesion plots might be used together with the present model to aid interpretation of (experimental) TEM images. This, we think, provides both a consistency check as well as additional worthwhile information.

Due to the local character of the MC steps, we can, albeit in a rough sense, relate the flocculation kinetics to the number of MC steps. The present simulations are for systems containing three components, i.e., elastomer, filler, and coupling agent. We would like to stress that, to the best of our knowledge, this is one of the first attempts to develop a theoretical approach to the modelling of the filler network morphology inside elastomers based on interface free energies, monitoring structural development in terms of simulated TEM images and SAXS intensities. The only related studies, which we are aware of, is Ref. [28], which does not discuss SAXS intensities, and work by Schneider [13], who calculates SAXS intensities for elastomer nano-composites based on simple cluster-cluster aggregation without specific interactions.

A limitation of the approach is its current restriction to a single rubber component. Usually, the experimental studies focus on polymer blends. This and a lack of information regarding the relevant surface tensions in most of the experimental work listed above, i.e., [13,14,15,16,17,18,19,20,21,22,23], currently imposes severe limitations in terms of experimental results to compare to. Note that the authors of Ref. [19] also point out the need for more experimental work on simple model systems and in fact do mention this as part of their motivation.

In principle, addition of extra components to our model is straightforward. A second type of elastomer is added easily via an extra type of cube in addition to the already existing types ‘rubber’ and ‘filler’. The entire approach is computationally cheap, unless the goal is the large scale network structure—here characterised in terms of a mass fractal dimension. If the initial aggregation behaviour is sufficient, then the approach is particularly suited for screening studies.

The thermodynamic model presented here is not an active mechanical model. This means that it does not yield the dynamic moduli. However, the standard monitoring of flocculation kinetics is based on measurements of the latter. Another extension, which we currently pursue, is the combination of this thermodynamic structure model with a previously developed dynamical model based on a similar type of coarse-graining (cf. Refs. [44,45,46,47]). This then allows the transfer of filler network configurations, generated at different ‘times’ along the MC trajectory, to the aforementioned dynamic model, in order to obtain the attendant dynamic moduli.

There is yet another use of the model worth mentioning. An improved physical description of the Payne effect, i.e., the pronounced decrease of the storage modulus with increasing strain amplitude, at least in some models, requires information on the number of reversible filler-to-filler contacts inside the network and, more precisely, the number of reversible filler-to-filler contacts along the load-bearing paths [31]. The current thermodynamic structure model in conjunction with the above mapping to a dynamical coarse-grained model can help to obtain the distribution of load-bearing network paths at a particular state of deformation as well as the aforementioned number of reversible filler-to-filler contacts.

## Abbreviations

The following abbreviations are used in this manuscript:

SA(X)S	small angle (X-ray) scattering
TEM	transmission electron microscopy
MC	Monte Carlo
BR	polybutadiene rubber
CR	polychloroprene rubber
NR	natural rubber
SBR	styrene-butadiene rubber
XNBR	carboxylated acrylonitrile-butadiene rubber
PI	polyisoprene
TESPT	Bis[3-(triethoxysilyl)propyl]tetrasulfide
